# Process Simulation and Techno-Economic Analysis of Large-Scale Bioproduction of Sweet Protein Thaumatin II

**DOI:** 10.3390/foods10040838

**Published:** 2021-04-12

**Authors:** Kirolos D. Kelada, Daniel Tusé, Yuri Gleba, Karen A. McDonald, Somen Nandi

**Affiliations:** 1 Department of Chemical Engineering, University of California, Davis, CA 95616, USA; kkelada@ucdavis.edu (K.D.K.); kamcdonald@ucdavis.edu (K.A.M.); 2 DT/Consulting Group, Sacramento, CA 95818, USA; daniel@dt-cg.com; 3 Nomad Bioscience GmbH, 06120 Halle, Germany; gleba@nomadbioscience.com; 4 Global HealthShare® Initiative, University of California, Davis, CA 95616, USA

**Keywords:** thaumatin, sweet protein, molecular farming, natural sweeteners

## Abstract

There are currently worldwide efforts to reduce sugar intake due to the various adverse health effects linked with the overconsumption of sugars. Artificial sweeteners have been used as an alternative to nutritive sugars in numerous applications; however, their long-term effects on human health remain controversial. This led to a shift in consumer preference towards non-caloric sweeteners from natural sources. Thaumatins are a class of intensely sweet proteins found in arils of the fruits of the West-African plant *Thaumatococcus daniellii*. Thaumatins’ current production method through aqueous extraction from this plant and uncertainty of the harvest from tropical rainforests limits its supply while the demand is increasing. Despite successful recombinant expression of the protein in several organisms, no large-scale bioproduction facilities exist. We present preliminary process design, process simulation, and economic analysis for a large-scale (50 metric tons/year) production of a thaumatin II variant using several different molecular farming platforms.

## 1. Introduction

The overconsumption of nutritive (caloric) sugars continues to be a major dietary problem in different parts of the world. A recent report indicates than an average American consumes about 17 teaspoons of added sugar daily, which is nearly twice the amounts of the 6 and 9 teaspoons, recommended for women and men, respectively [1]. This dietary behavior is linked to various adverse health effects such as increased risk of diabetes, obesity, high blood pressure and cardiovascular diseases [2]. Hence, there are worldwide efforts to reduce sugar consumption. For instance, the World Health Organization (WHO) made a conditional recommendation to reduce sugar consumption to less than 5% of the total caloric intake, along with a strong recommendation to keep sugar consumption to less than 10% of the total caloric intake for both adults and children [3]. Currently, added sugar consumption accounts for approximately 11–13% of the total energy intake of Canadian adults [4], is greater than 13% in the US population [5], and is as high as 17% in US children and adolescents [6], the latter principally from sugar-sweetened beverages (SSB). Consequently, taxes on SSB have been proposed as an incentive to change individuals’ behavior to reduce obesity and improve health [7]. Notably, the city of Berkeley, CA, USA successfully accomplished a 21% decrease in SSBs consumption within a year of implementation [8]. Therefore, it is expected that more states and cities will adopt this policy. On the regulatory level, the U.S. Food and Drug Administration (FDA) updated the Nutrition Facts label requirement on packaged foods and beverages, starting 1 January 2020, to declare the amount of added sugars in grams and show a percent daily value for added sugar per serving. Serving sizes have also been updated to reflect what people currently eat and drink [9]. The expansion of these efforts to spread the awareness on sugar consumption habits and the resulting health issues has generated demand for safe, non-nutritive (low/zero calorie) sugar substitutes. There are many sweeteners on the market to help consumers satisfy their desire for sweetness; however, each of the sweeteners available to consumers has specific applications and certain limitations [10].

Artificial sweeteners (ATS) have been used as sugar substitutes in numerous applications; however, their long-term effects on human health and safety aspects remain controversial [2]. For example, ATS appear to change the host microbiome, lead to decreased satiety, alter glucose homeostasis, and are associated with increased caloric consumption and weight gain [11]. Moreover, some health effects such as dizziness, headaches, gastrointestinal issues, and mood changes are associated with the consumption of a commonly used ATS, aspartame [12]. Additionally, Kokotou et al. [13] have demonstrated the impact of ATS as environmental pollutants, concluding that when artificial sweeteners are applied in food products or eventually enter the environment, their transformation and/or degradation may lead to the formation of toxic substances. Consequently, there is currently an increase in the production of natural sugar alternatives based on the shift in consumer preferences toward more natural products to meet their dietary need and restrictions [14].

Stevia, the common name for glycoside extracts (rebaudioside A and stevioside) from the leaves of *Stevia rebaudiana*, is a natural, sweet-tasting calorie-free botanical that is currently gaining popularity as a sugar substitute or as an alternative to artificial sweeteners [15]. Recent reports project the annual growth rate of stevia compounds to be 6.1% and 8.2%, during 2015–2024 and 2017–2024, respectively [16]. Stevia has gained industry acceptance in recent years due to its ease of cultivation in several countries across the globe [16] and its high sweetness index (160–250 times sweeter than sucrose [17]). This shows that the growth of stevia’s use as a sugar substitute, despite taste limitations of the marketed glycosides [18], was contingent on the feasibility of its large-scale manufacturing.

Thaumatin, monellin, manbinlin, pentadin, brazzein, curculin, and miraculin are sweet tasting proteins that are naturally expressed in tropical plants. Studies have found that human T1R2-T1R3 receptors expressed in taste buds in the mouth and (also in a variety of non-taste organs) recognize natural and synthetic sweetness while T1R1-T1R3 recognize the umami (savory) taste [19]. These receptors, which have several binding sites [20], are activated when the compounds that elicit sweet taste bind to them. However, these proteins have unique binding properties and do not all bind at the same sites [21], which leads to varying perception of sweetness. This work focuses on thaumatins, a class of intensely sweet proteins isolated from the arils of the fruits of the West-African plant *Thaumatococcus daniellii* [22]. The distinctiveness of thaumatin lies in its sweetness index being up to 3500 times sweeter than sugar [17]. According to the 2008 Guinness World Records, it is the sweetest natural substance known to mankind [23]. Thaumatin I and II, the two main variants of the protein, are comparable in their biological properties, structure, and amino acid composition. The structure consists of a single polypeptide chain of 207 amino acids that are linked together by 8 disulfide bonds [2]. The two variants differ by only five amino acid residues. Through chemical modifications and site-directed mutagenesis, it has been determined that the residues on the cleft-containing side of the protein have the strongest effect in eliciting sweetness to taste receptors on the tongue [24]. The specificity of these residues demonstrates the importance of the protein structure in inducing thaumatin’s sweetness.

In the USA, extracted thaumatin and thaumatin B-recombinant were initially affirmed Generally Recognized as Safe (GRAS) flavor enhancers/modifiers (FEMA no. 3732 and 3814, respectively) [25], but not as sweeteners. In the USA, plant-made (in food species) thaumatin I and/or thaumatin II were granted GRAS status by the FDA in 2018 for use as a sweetener (GRN 738). In 2020, the FDA granted GRAS status to recombinant thaumatin II produced in *Nicotiana* plants for use as a sweetener (FDA GRAS GRN 910) and as a flavor enhancer/modifier (FDA GRAS GRN 920). In the EU, thaumatins are allowed as both sweeteners and flavor enhancers (E 957) [17,26]. Thaumatin’s safety has been extensively documented. The Joint FAO/WHO Expert Committee on Food Additives (1986) report claims that the protein is free from any toxic, genotoxic, or teratogenic effects [27]. Thaumatin is currently used as a flavor modifier in food applications such as ice creams, chewing gum, dairy, pet foods, soft drinks, and to mask undesirable flavor notes in food and pharmaceuticals [2]. 

The current top global thaumatin manufacturers are Naturex (Givaudan), France; Beneo Palatinit, Germany; Natex, UK and KF Specialty Ingredients, Australia. The global production of thaumatin increased to 169.07 metric tons (MT) in 2016 from 138.47 MT in 2012 [28]. However, the current production method through aqueous extraction from the fruits of the tropical plant *T. daniellii* limits its availability while the demand is increasing [2]. *T. daniellii* is not cultivated and harvesting of the arils takes place in plants growing wild in rainforests of West Africa ranging from Sierra Leone to the Democratic Republic of Congo. The current production process is substantially dependent on the availability and quality of the native plant from year to year, which limits thaumatin’s use as a commodity (sweetener) product. 

The emergence of recombinant DNA technology and the use of cultured cells have allowed the production of proteins in large quantities. Enzymes (proteases, lipases, amylases, etc.) and structural proteins are used in many industrial applications including the production of food and beverages, biodiesel, cosmetics, biopolymers, cleaning materials, and waste management [29]. Most importantly, recombinant production allows for the expression of a protein outside its native source. Therefore, there exists a viable alternative to secure the desired quantities of thaumatin reliably and sustainably, without impacting rainforest ecosystems. Notably, there have been many attempts to produce thaumatin by means of genetically engineered microorganisms and plants. Despite successfully expressing thaumatin in yeast [30], bacteria [31], fungi [32], and transgenic and transfected plants [33], biotechnological large-scale production facilities have yet to be established [17]. 

Molecular farming, the production of recombinant proteins in plants, offers several advantages over bioreactor-based systems. In this application, plants are thought of as nature’s single use bioreactors, offering many benefits such as reduced upstream production complexity and costs (due to less expensive infrastructure and raw materials), linear scalability, and their inability to replicate human viruses [34]. Specifically, open-field growth of plants has the potential to meet the market’s need for a large-scale, continuous demand of a commodity product at a competitive upstream cost. It has been marked suitable for this operation as plants can be easily adapted on an agricultural scale to yield several metric tons of the purified protein per year [35]. Here, we present a feasibility study for a protein production level of tens of metric tons per year.

The success of a new product in the biotechnology process industry depends on well-integrated planning that involves market analysis, product development, process development, and addressing regulatory issues simultaneously, which requires some decisions to be made with limited information [36]. This generates demand for a platform to help fill in those gaps and facilitate making more informed process and technology decisions. Process simulation models (PSMs) can be used in several stages of the product life cycle including idea generation, process development, facility design, and manufacturing. For instance, based on preliminary economic evaluations of new projects, they are used to eliminate unfeasible ideas early on. During the development phase of the product, as the process undergoes frequent changes, such models can easily evaluate the impact of these changes and identify cost-sensitive areas. PSMs are also useful for directing lab and pilot-scale studies into areas that require further optimization. Additionally, PSMs are widely used in designing new manufacturing facilities mainly as a tool for sizing process equipment and supporting utilities, as well as for estimating the required capital investment and cost of goods. This ultimately helps companies decide on building a new facility versus outsourcing to contact manufacturers [37]. 

There are currently few published data-driven simulations of techno-economic models for plant-based manufacturing of proteins for pharmaceutical [38,39,40,41,42], biofuel [40], commercial enzyme [43], and food safety applications [44]. However, to the best of our knowledge, no studies have proposed or assessed the feasibility of plant-based protein bioproduction platforms on the commodity scale in tens of metric tons per year. The feasibility of production at this scale is critical for the emergence of thaumatin as a sugar substitute. Here, we present a preliminary process design, process simulation, and economic analysis for the large-scale manufacturing of thaumatin II variant (will be referred to as thaumatin for the rest of the report) by several different molecular farming production platforms.

## 2. Materials and Methods

### 2.1. Process Simulation and Economics

Process simulation and economic analysis of all scenarios was performed using SuperPro Designer^®^ (“SuperPro”) Version 10 build 7 (Intelligen, Inc., Scotch Plains, NJ, USA; http://www.intelligen.com). The software allows modeling of various widely used unit operations. However, some unit operations and processes used in this study that are not included in SuperPro’s suite, such as field cultivation, mechanical harvesting, and screw press extraction. This was addressed by using the “Generic Box” feature of the software. SuperPro was used in equipment sizing, performing mass and energy balances, batch scheduling/debottlenecking, and Capital Expenditure (CAPEX), Operating expenditure (OPEX), and cost of goods (COGS) calculations. The model inputs are process and economic parameters obtained using working process knowledge, unpublished lab or pilot scale data from the author’s laboratories, published data from literature, and/or SuperPro’s built-in values. The Base Case SuperPro model developed in this study is publicly available and can be downloaded from the following website: https://mcdonald-nandi.ech.ucdavis.edu/tools/techno-economics/. A free trial version of SuperPro (http://www.intelligen.com/demo.html) can be used to view the model.

### 2.2. Base Case Process Assumptions

The base case scenario assumes an annual production capacity of 50 MT thaumatin. To achieve this level of production in a consistent manner, manufacturing is divided into 157 annual batches. Upstream production is attainable through open-field, staggered plantation of *Nicotiana tabacum* plants. Each batch has a duration of 45 days (including land turnaround) and a recipe cycle time (the amount of time between the start or end of two consecutive batches) of 2 days. A full list of process assumptions can be found in Table S1.

The proposed design achieves the expression of thaumatin in *N. tabacum* leaves using magnICON^®^ v.3. This technology developed by Icon Genetics GmbH (Halle/Saale, Germany) allows for the separation of the “growth” and the “expression” phases in a manufacturing process. Moreover, this process obviates the need to use agroinfiltration, which requires more capital and operational costs for inoculum preparation and implementation of expensive units for the infiltration process, containment of the genetically engineered agrobacteria, and elimination of bacteria-derived endotoxins [45,46]. In this design, transgenic *N. tabacum* or *N. benthamiana* plants carry a double-inducible viral vector that has been deconstructed into its two components, the replicon and the cell-to-cell movement protein. Background expression of recombinant proteins prior to induction remains minimal; however, inducible release of viral RNA replicons—from stably integrated DNA proreplicons—is triggered upon spraying the leaves and/or drenching the roots with a 4% (*v/v*) ethanol solution resulting in expression levels as high as 4.3 g/kg fresh weight (FW) in *Nicotiana benthamiana* [46]. Nonetheless, *Nicotiana tabacum* (tobacco) has several advantages that make it more suitable for large-scale open field production such as field hardiness, high biomass yields, well-established infrastructure for large-scale processing, plentiful seed production, while attaining expression levels up to 2 g/kg FW [47,48]. Furthermore, it is unlikely that transgenic tobacco material would mix with material destined for the human food or animal feed chain, unless it is grown in rotation with a food crop, but further development of strict Good Agricultural Practice for transgenic plants should overcome these issues [47].

### 2.3. Indoor Vertical Farming

An alternative upstream facility design scenario was developed to evaluate the process economics of a more controlled supply of thaumatin by growing the plant host in a 10-layer vertical farming indoor environment. *Nicotiana benthamiana* is chosen as a host because it is known to be a model for protein expression for both *Agrobacterium* and virus-based systems, but its low biomass yield and difficulties regarding adaptation in the field hinder its application for open outdoor growth. However, this species grows very well in indoor, controlled environments and has high recombinant protein production. This upstream production facility uses the same method of expression and follows the same schedule as the base case upstream facility. 

### 2.4. Transient Production in Spinach

Transient expression in plants is a method of recombinantly producing proteins without stable integration of genes in the nuclear or chloroplast genome [49]. The main advantages of using this method are reducing the extensive amount of time needed to develop a stable transgenic line and overcoming biosafety concerns with growing transgenic food crops in the field expressing heterologous proteins [50]. Transient expression is attainable through several systems including biolistic delivery of naked DNA, agrobacteria, and infection with viral vectors. Notably, the use of viral vectors has been marked suitable for application on a field-scale due to the flexibility of production, and the quick accumulation of target proteins while achieving high yields [51,52,53]. A new report [54] has shown efficacy in delivering RNA viral particles using a 1–3 bar pressure, 1–4 mm atomizer nozzles spray devices in the presence of an abrasive to cause mechanical wounding of plant cell wall. 

GRAS notices GRN 738 and GRN 910 describe production of thaumatin in edible plant species and *N. benthamiana*, respectively. The expression of thaumatin in leaf tissue of the food crops *Beta vulgaris* (beet), *Spinacia oleracea* (spinach), or *Lactuca sativa* (lettuce) (GRN 738) is generally lower than in *N. benthamiana*. However, despite having lower expression levels, the absence of pyridine alkaloids (e.g., nicotine) that are present in *Nicotiana* species is a major advantage for production in food crops because of the significant downstream resources needed to remove alkaloids in *Nicotiana*-based products [44]. The ultimate solution may be a high-expressing engineered *Nicotiana* host devoid of alkaloid biosynthesis [55], but that option was not modeled in this study. 

The transient production facility is designed to produce 50 MT of purified thaumatin in spinach, annually, over 153 batches due to longer turnaround time required for *S. oleracea* compared to *N. tabacum* crops. Each batch has a duration of 67.8 days and a recipe cycle time of 1.94 days. 

## 3. Results

### 3.1. Field Upstream Transgenic Production Facility

**Figure 1:**
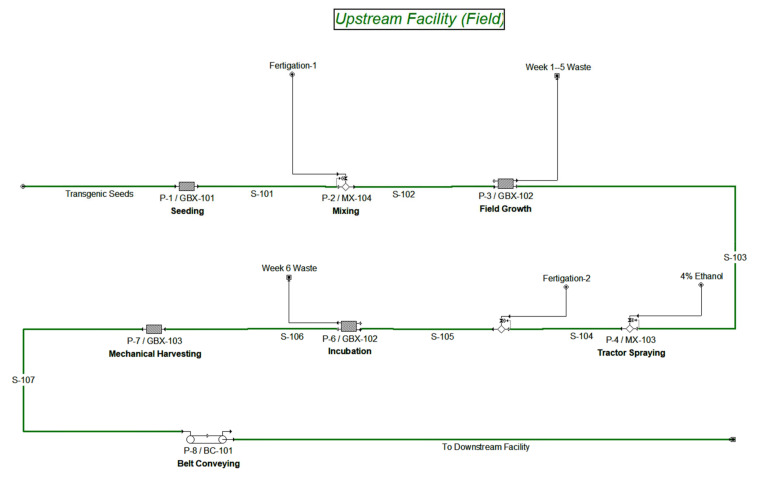
SuperPro Designer model flowsheet for base case upstream transgenic production facility.

The proposed base case upstream field production facility, displayed in Figure 1, consists of a 540 acre block of land divided into 22 plots, each of which is suitable for growing 318,000 kg FW of *N. tabacum*, carrying 477 kg of thaumatin, accounting for downstream recovery of 66.8%. It is assumed that the facility is located in a suitable climate where the growth of *N. tabacum* is attainable throughout the year, ignoring variations in production between batches (e.g., all batches are assumed to be identical). Each batch starts with direct seeding of transgenic *N. tabacum* plants in the field (GBX-101). The seeds are left to germinate for two weeks followed by vegetative growth for 3 more weeks post germination (GBX-102). A fertigation stream is applied to deliver the necessary nutrients for optimal plant growth. After a total of 35 days post seeding, a tractor sprayer (MX-103) applies 4900 L of a 4% (*v/v*) ethanol solution [40] to the plot’s crop, triggering the synthesis and accumulation of thaumatin in plant biomass. The plants are incubated for 7 more days, during which time they continue to uptake nutrients and express thaumatin. After 42 days from seeding, the batch is harvested through two mechanical harvesters and four hopper trucks (GBX-103) at a rate of 17,000 kg/h and transported to downstream processing facility using a conveyer belt (BC-101). The plot undergoes a turnaround period of three days for which the labor and equipment cost is included. No pesticides, fungicides, or herbicides costs are added due to the assumption that not enough growing degree days are accumulated during the batch cycle duration (42 days), for disease-causing organisms to be a concern. 

### 3.2. Indoor Upstream Transgenic Production Facility

Transgenic *Nicotiana benthamiana* seeds are germinated in soilless plant substrate at a density of 94 plants per (30 cm × 50 cm) tray (GBX-101). Seedlings are then grown hydroponically, under LEDs, until reaching manufacturing maturity after 35 days. Induction occurs in a separate hydroponic reservoir, where plants are root drenched and sprayed with 0.01 L of 4% (*v/v*) ethanol per kg FW plant tissue. The plants are left to grow for 7 additional days during the incubation period. *N. benthamiana* biomass is mechanically harvested (GBX-103) at a rate of 54,000 kg/h and transported to downstream processing facility using a conveyer belt (BC-102). The indoor upstream facility flowsheet can be found in Figure S1. The total facility footprint was calculated to be 83,000 m^2^.

### 3.3. Transgenic Production Downstream Processing Facility

**Figure 2:**
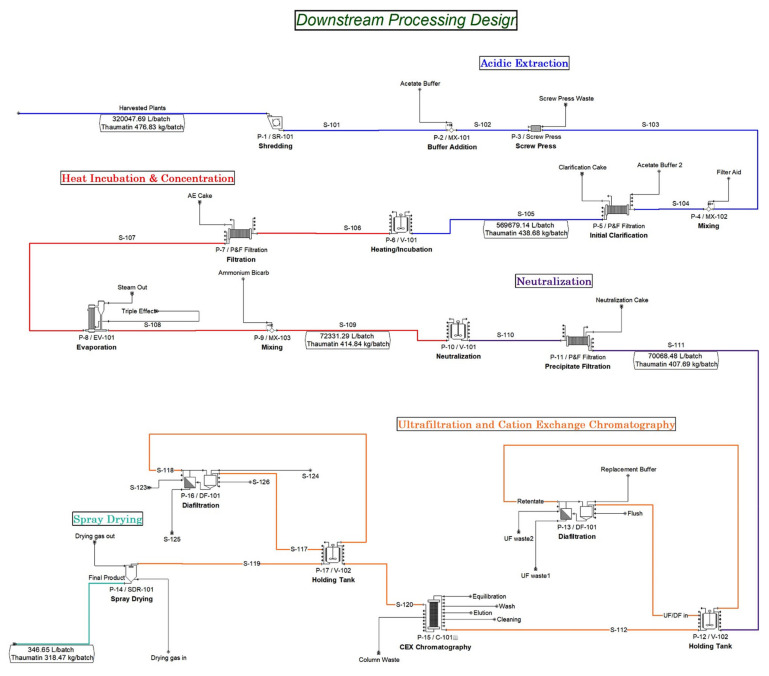
SuperPro Designer model flowsheet for base case downstream processing facility.

The base case downstream processing (DSP) facility (Figure 2) is designed to purify and formulate 318.5 kg/batch of thaumatin with 98% purity. A DSP batch starts with shredding plant biomass using two industrial shredders (SR-101), each processing 40,000 kg of plant biomass/h. This step is designed to homogenize the leaves and stems to facilitate the extraction process. Shredded plant material is then mixed with an acetate buffer in a 0.8 L of buffer to 1 kg of biomass ratio. This step leverages stability of thaumatin at low pH (2.7 to 6.0) [2] to precipitate host plant proteins that aren’t stable under acidic conditions. The extraction buffer consists of 50 mM acetic acid and 150 mM sodium chloride mixture at a pH of 4.0. The resulting plant slurry is then fed into a screw press to separate most of the dry plant material. A screw press is recommended for this step because it minimizes the amount of extraction buffer needed by forcing out more plant sap with the increasing pressure inside the chamber [56]. The crude extract stream obtained from the screw press unit is sent to three parallel P&F filtration units for initial clarification, each having a membrane area of 190 m^2^. Furthermore, the model assumes the use of food-grade filter membranes designed to include 10 filter sheets with decreasing particle retention size from 25 to 0.1 μm. The acetate buffer is applied once again as cake wash with a 0.2 L buffer to 1 L extract ratio. Diatomaceous earth is added to this step as a filter aid in a 6:100 (wt diatomaceous earth:wt biomass) [38]. The stability of thaumatin at low pH and high temperatures facilitates the precipitation of more host cell proteins as well as other undesired plant-derived compounds. Using seven heating tanks (76,500 L/tank, assuming 90% maximum allowable working volume), the plant extract is then heated to 60 °C for 60 min. Following heat incubation, the stream is sent to a P&F filtration unit to capture the heat-precipitated proteins. It is assumed that a 90% reduction of *N. tabacum* total soluble proteins is attainable following the heat incubation and precipitation steps [57]. Concentrating the thaumatin stream prior to the ultrafiltration/diafiltration (UF/DF) step is necessary to avoid processing large liquid volumes ~573,000 L further downstream. It has been reported that thaumatin experiences a loss in sweetness when heated above 70 °C at a pH of 7.0 [2]; therefore, the product stream undergoes concentration by evaporation prior to neutralizing the solution since the protein can sustain higher temperatures at a low pH [2].The triple effect evaporation unit (EV-101) is designed to evaporate 90% of the water content in the stream at 109 °C, 77 °C, and 40 °C in the first, second, and third effect, respectively, over 4 h. 

The exiting stream (S-108) is then neutralized with 1:1 (ammonium bicarbonate:acetic acid) molar ratio and mixed in V-101 for 30 min and sent to the P&F filtration unit to remove any precipitated materials. An additional 1.5% loss of thaumatin during this step is assumed. Because soluble impurities such as nicotine and other pyridine alkaloids are abundant in *N. tabacum* plants, a UF/DF step is necessary to eliminate small molecules. The UF/DF unit consists of 4 stacked cassette holders, each containing twenty 3.5 m^2^ cassettes. Since thaumatin is a 22 kDa protein, a membrane with MWCO of 5 kDa is used per working process knowledge. Assuming a conservative flux of 30 L/(m^2^ h), the inlet stream is concentrated using a concentration factor (CF) of 5, diafiltered 10 times against reverse osmosis (RO) water, then re-concentrated using a CF of 5 over 20.6 h, resulting in a 75% pure thaumatin and nicotine content of 1.08 mg/kg thaumatin. A retention coefficient of 0.9993 was assumed for thaumatin, resulting in 5.8% thaumatin loss in UF/DF (working process knowledge). The retentate is then sent to five CEX chromatography columns operating in parallel (520 L bed volume each) which was modeled based on unpublished data from Nomad Bioscience GmbH (Halle, Germany). GE Healthcare Capto S resin with an assumed binding capacity of 150 g/L was used in this analysis. Table S2 shows the downstream losses breakdown per unit operation. Spray drying is used as a final formulation step over other means of industrial drying due to the heat sensitivity of thaumatin. 

### 3.4. Spinach Transient Production Facility

The simulated facility (Figure S2) consists of three sections—Virion production laboratory (VPL), spinach field growth, and DSP. A list of base case design parameters and assumptions is shown in Table S3. The VPL process is adopted from a recent article [54] entailing the production of RNA viral particles (infective virions) from agrobacteria carrying a PVX construct. The laboratory is sized to produce 7900 L of spray solution per batch for application in the field. *Nicotiana benthamiana* plants are used as the host to produce the viral particles to inoculate spinach. *N. benthamiana* seeds are germinated (GBX-101) in soilless plant substrate at a density of 94 plants per (30 cm × 50 cm) tray. Seedlings are grown hydroponically (GBX-102), under LEDs, until reaching manufacturing maturity at day 35. *Agrobacterium tumefaciens* is grown for 24 h, before being left in a 4 L flask (SFR-101) overnight, and the *A. tumefaciens* suspension is added to MES buffer in V-101. *N. benthamiana* infiltration takes place in a vacuum agroinfiltration chamber (GBX-103) for 24 h followed by incubation for 7 days in (GBX-104). *N. benthamiana* biomass production, agrobacterium growth, agroinfiltration, and incubation parameters are adapted from [38]. After the incubation period, 41.5 kg of *N. benthamiana* fresh weight (FW) are ground (GR-101) and mixed with PBS buffer in a 5:1 (*v/w*) buffer:biomass ratio. The extract is then sent to a decanter centrifuge (DC-101) to separate plant dry matter from the liquid phase which is clarified by dead-end filtration (DE-101), followed by mixing the permeate with 35.9 kg of diatomaceous earth and 7780 L of water to reach a final concentration of 10^14^ viral particles/L and 4.55 g diatomaceous earth/L. Diatomaceous earth is used as an abrasive to mechanically wound plant cell walls allowing the virions to enter the cytoplasm of the cell [58]. The final spray is stored in (V-102) for 13 h before field application. 

Field operation starts at the beginning of each batch with the direct seeding (GBX-201) of 28.3 million *Spinacia oleracea* seeds (14.2 kg) over 22.6 acres [59]. Spinach is planted over 80-inch beds with an assumed 3 ft spacing between beds, resulting in 14,520 linear bed feet per acre [60]. Seeds are germinated and grown in the field for 44.5 days, during which time a drip irrigation system (MX-201) delivers irrigation water and soluble fertilizer (fertigation) to the soil. It is assumed that 200 acre-inches of irrigation water and 64 tons of fertilizer are needed per batch [61]. A tractor on which multiple high-pressure spray devices are mounted (GBX-203) is used to deliver the viral particle solution at a rate of 2 acres/h [52]. This method of delivery has shown high effectiveness (>95% infection of treated plants) [52]. Spinach plants are incubated in the field for 15 days post-infection. During that period, thaumatin starts to accumulate in the crop at an average expression level of 1 g/kg FW after 15 days post-spraying. At day 60, two mechanical harvests (GBX-204) collect a total of 344 MT spinach biomass, carrying 344 kg thaumatin, with the aid of four hopper trucks, which is transferred to a 500-m-long conveyor belt (BC-201) that extends from the field collection site to the DSP section of the facility. Harvesting occurs at an average rate of 17,000 kg FW/h, which is estimated based on a harvester speed of 5 km/h and 14,520 linear bed feet per acre [60,62]. 

A more simplified downstream processing, enabled by the use of spinach as a host, starts with mixing plant material with 65 °C water (MX-301) before extracting the green juice (GJ) through a screw press (GBX-301). The resulting GJ is heated for 1 h at 65 °C in ten jacketed tanks (V-301), then concentrated by evaporation (EV-301) to reduce product stream volume for further purification steps. Since thaumatin is not stable at temperatures above 70 °C at neutral pH [2], evaporation is performed at a low temperature of 40 °C and 0.074 bar vacuum pressure [63]. Thermally degraded host cell proteins and impurities are eliminated in a P&F filtration unit (PFF-301) designed to include 10 filter sheets with decreasing particle retention size from 25 to 0.1 μm. Smaller impurities are removed using a diafiltration unit (DF-301) with 5 kDa molecular weight cut off cassettes in a similar process as described in Section 3.3, the retentate is spray dried in (SDR-301) to obtain a final product which has 5% (*w/w*) water content, and 348 kg of solid material containing 94% pure thaumatin and 6% spinach impurities. These impurities are expected to be water soluble, heat stable molecules in the range of 5–100 kDa, according to the theoretical design of the filtration scheme.

### 3.5. Economic Analysis of Transgenic Production Facilities

We evaluated the CAPEX assuming a green-field project, including construction, validation, and start-up of new facilities for each production platform. The direct fixed capital (DFC) and working capital (WC) estimation parameters for all facilities are displayed in Tables S4–S6. Table 1 and Table 2 show the economic summary for transgenic and transient production, respectively. The annual operating costs (AOC) and cost of goods (COGS) were calculated for each section with and without depreciation, assuming depreciation over 10 years using the straight-line method, and a 5% salvage value. Our analysis shows that the inclusion of chromatography to improve the purity of *Nicotiana* made thaumatin adds $31 MM, $29 MM, and $1 MM to the CAPEX of downstream, upstream indoor, and upstream field facilities, respectively. The corresponding COGS (excluding depreciation) is increased by $240/kg, $320/kg, and $12/kg for downstream, upstream indoor, and upstream field facilities, respectively. 

**Figure 3:**
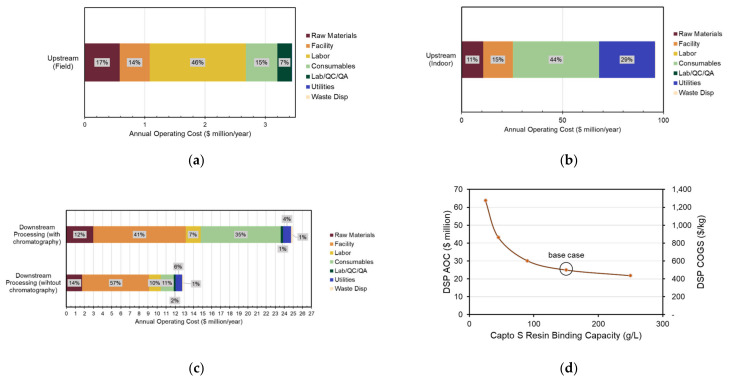
Annual operating costs breakdown per category for (**a**) field upstream transgenic facility, (**b**) indoor upstream transgenic facility, (**c**) base case downstream processing facility and without chromatography unit operation, (**d**) effect of resin binding capacity on DSP AOC and COGS. Depreciation costs are excluded. AOC, annual operating costs; COGS, cost of goods; DSP, downstream processing.

As shown in Figure 3a, field labor is the highest contributor to the upstream field facility followed by consumables. Detailed labor requirement and cost estimation calculations can be found in Tables S7 and S8. Consumables include mechanical harvester and tractor’s fuel, lubrication, and repair costs and other field equipment repair costs. Upstream indoor facility AOC breakdown (Figure 3b) elucidates a high cost of consumables due to the cost of soilless plant substrate, followed by high energy consumption from the LED lighting system used for plant growth. The labor category does not appear clearly on the chart because of the low need for labor hours since the indoor facility is highly automated.

In both DSP scenarios, facility-dependent costs have the highest cost impact. Insurance, local (property) taxes, and other overhead expenses are estimated to be 1%, 2%, and 5% of the section’s DFC, respectively [64]. Maintenance costs are also included in this category and estimated to be 10% of equipment purchase prices. Facility dependent cost estimation parameters are shown in Tables S9 and S10. Consumables account for 38% of the DSP facility with chromatography due to the high cost of Capto S resin ($1450/L) that is changed every 100 cycles. The effect of varying resin binding capacity to the product on the DSP AOC and COGS is shown in Figure 3d.

### 3.6. Expression Level and Production Capacity Scenario Analysis

Transgenic production models were resized based on scenario design requirement for production levels ranging from 10–150 MT and expression levels ranging from 0.5–2.5 g/kg, while keeping the scheduling parameters the same from base case models. 

**Figure 4:**
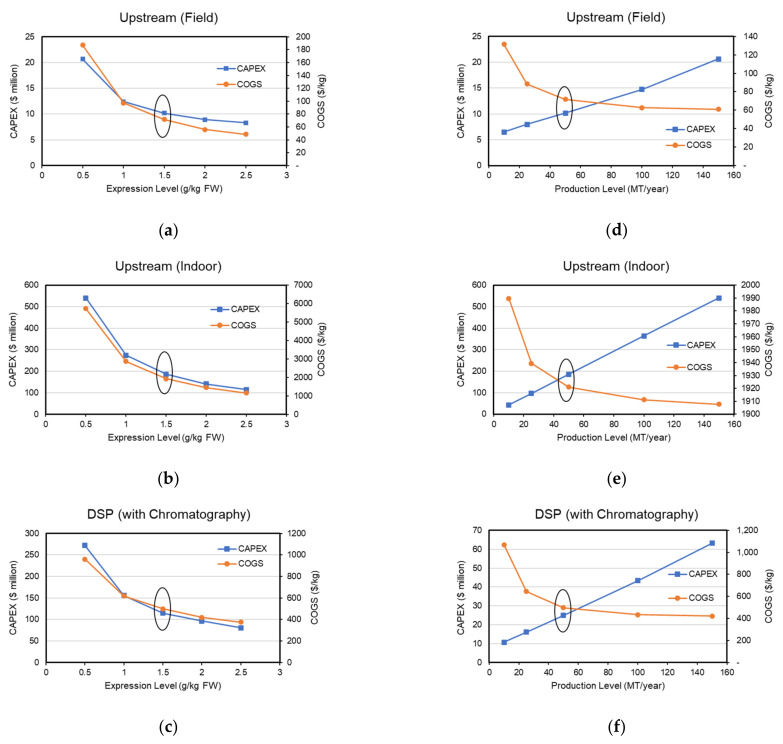
Scenario analysis varying expression level effects on CAPEX and COGS for (**a**) field upstream transgenic facility, (**b**) indoor upstream transgenic facility, (**c**) base case downstream processing facility. Scenario analysis varying production level effects on CAPEX and COGS for (**d**) field upstream transgenic facility, (**e**) indoor upstream transgenic facility, (**f**) base case downstream processing facility. Depreciation costs are excluded. Base case values are circled. CAPEX, Capital expenditure; COGS, cost of goods sold; DSP, downstream processing; FW, fresh weight.

The significant impact of expression level on CAPEX and COGS is elucidated in Figure 4a–c. Production level shows a very small decline in COGS for indoor upstream facility and a linear increase in CAPEX with increasing production level. On the other hand, the field upstream facility showed a significant increase in COGS at lower production levels due to the minimum ownership costs of field equipment regardless of the small acreage size. DSP followed the expected behavior that economy of scale dictates, with sharp decrease in COGS at lower production levels and diminishing returns at higher production levels. The deviation from linear trend at 150 MT/year in field upstream and DSP is likely due to the model’s specified equipment maximum rating, which allows for the inclusion of a new equipment in parallel beyond this rating.

### 3.7. Economic Analysis of Transient Production Facility

**Figure 5:**
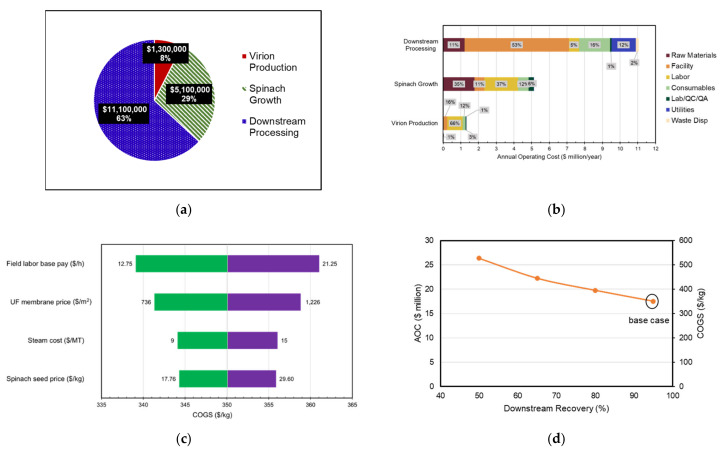
Spinach made annual operating cost breakdown (**a**) per facility section (**b**) section’s category. (**c**) Tornado analysis for facility’s top cost drivers. (**d**) Effect of varying downstream recovery on AOC assuming a constant target production level of 50 MT/year. AOC, annual operating costs; COGS, cost of goods sold; MT, metric tons.

As shown in Table 2, the DSP section of the facility accounts for 79% of the project’s CAPEX and 63% of AOC. This is justified by the high equipment purchase prices, piping, instrumentation, buildings, engineering, and construction costs for a plant of this size. Figure 5a shows field labor as the highest cost contributor to the spinach field growth section due to the high direct demand of 48,800 labor-h/year, followed by the cost of spinach seeds, which is estimated to be $23.68/kg for the leafy Bloomsdale variety. Mechanical harvester and tractor’s fuel, lubrication, and repair costs are in included as consumables as well as other field machinery repair costs. Due to the small-scale scope of the VPL, labor is the highest contributor of the section’s operating cost.

The impact of varying the highest cost drivers in each of the facility’s category (field labor rate, labor; UF membrane, consumables; steam cost, utilities; spinach seeds, raw materials) by 25% on COGS is portrayed as a tornado diagram in Figure 5c. Field labor was the most sensitive cost variable, having the highest impact on the COGS, followed by the ultrafiltration (UF) membrane, which is replaced every 30 cycles. 

In this model, we assume a relatively high downstream recovery (95%) of the protein from harvest to formulation. The reason for this assumption is that spinach, being edible crop, allows for a lower target product purity (as long as the sensory profile of thaumatin is unaffected) and a consequently fewer DSP steps. It is particularly important to focus resources on maximizing downstream recovery during process development because it ultimately affects plant biomass and spray volume requirement upstream to appropriately compensate for these losses, which in turn affects equipment sizing in DSP based on the amount of plant material to be processed. The unit operations were resized according to the scenario design requirement for downstream recovery ranging from 50 to 95% while scheduling parameters were left unchanged. This effect of downstream recovery on the facility’s AOC and COGS is shown in Figure 5d and shows a 1.5× increase in AOC and COGS as downstream recovery decreases from 95% to 50%. 

## 4. Discussion

**Figure 6:**
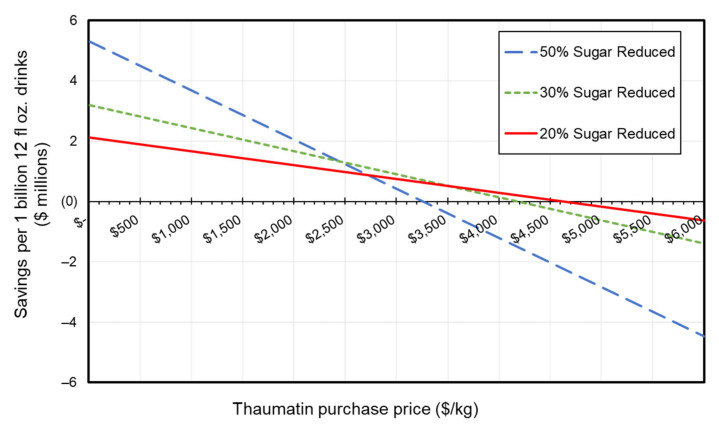
Potential cost savings from thaumatin II use as a function of its purchase price. Tested scenarios of 20%, 30%, and 50% sugar reduction in 10% (*w/v*) sugar sweetened soft drinks assuming a $0.30/kg price of sugar. Thaumatin replacement quantities calculated using a published sensory regression analysis (FDA GRAS GRN 910).

Although our analysis indicates a relatively high COGS range for a sugar substitute, there are unrealized costs savings from thaumatin use due to its unique sweetness intensity. Thaumatin’s use in extremely small quantities is essentially why it is considered a non-caloric sweetener, as it provides only 4 calories per gram. Sensory evaluation studies have found that a sample with 5% (*w/v*) sucrose +4.6 ppm thaumatin II had similar sweetness as a 10% sucrose control with minimal lingering aftertaste, suggesting that up to one-half of the sugar could be replaced by thaumatin II (FDA GRAS GRN 910). SSBs including sodas, fruit drinks, and sport drinks account for 50% of the total added sugar in Western diets [65], and therefore provide an attractive avenue for thaumatin emergence as a sugar substitute. The incorporation of thaumatin by the industry not only offers a tool to help decelerate the obesity epidemic caused by increased childhood sugar intake decades ago [66], but also provides itself with a more economically viable solution. Firstly, as sugar taxations emerge, sugar reduction becomes a financial incentive. Secondly, the reduction of sugar and the addition of thaumatin to retain the same level of sweetness has the potential to save millions of dollars per day on the cost of sweetening beverages. Assuming that the average “standard” sucrose concentration in SSBs is 35.5 g per 12 fl oz. drink ~10% (*w/v*) [67], and a $0.30/kg sugar price, Figure 6 shows the potential savings from using thaumatin to reduce sugar content by 20%, 30%, and 50%, while maintaining the same sweetness as the standard for a range of thaumatin purchase prices. The amount of thaumatin needed to obtain the same sweetness as a 10% solution in each sugar reduction scenario was calculated using the sensory regression analysis included in a published GRAS notice (FDA GRAS GRN 910). Table 3 shows the daily and annual amount of thaumatin needed for each sugar reductions scenario, assuming that one billion 12 fl oz drinks are to be sweetened per day. Successful implementation of thaumatin in this avenue can liberate R&D resources to improve expression levels and increase production volumes, both of which have a substantial impact on COGS reduction, as we have demonstrated. 

Our preliminary engineering facility design indicates the feasibility of thaumatin manufacturing by various molecular farming platforms. The most economic method (based on COGS, without accounting for depreciation) is the field grown ethanol-inducible, transgenic *N. tabacum*, assuming a downstream facility without chromatography (COGS: $318/kg). It remains unclear whether heat incubation is sufficient to achieve the desired purity for a safe product without the inclusion of chromatography on a large-scale. In a previous plant-made food safety product techno-economic analysis [44], a chromatography unit was included for protein purification from *N. benthamiana*; however, heat precipitation of host cell proteins was not included as a purification step. We also demonstrate the importance of resin selection and thorough chromatography operation optimization by evaluating the cost benefit of maximizing resin binding capacity to target product. Of course, further work is needed to verify whether the use of column chromatography is needed.

Transient production of thaumatin in the edible crop *Spinacia oleracea* was also economically competitive (COGS: $350/kg) and captures the benefits of obviating the need for an intensive DSP. According to this analysis, the cost to produce a kg of fresh weight (FW) of spinach is $0.10, as opposed to a cheaper price for tobacco ($0.07/kg FW). This is attributed to the higher cost of the seeds of spinach, the longer turnaround time assumed for spinach, and the higher plant density assumed for tobacco. It is evident that field operation is very labor intensive, due to the low recipe cycle time of 2 days, which is different than the traditional timeframe of growing those crops.

The potential for high intra-batch variations in product yield and quality due to meteorological factors is one of the concerns of using field grown plant material for this application. These variations in turns cause inconsistency in key facility performance parameters that should be quantified using a probabilistic approach and communicated to stakeholders and will be addressed in a follow-up communication. The cost of obtaining a more controlled supply of product is reflected in the indoor upstream facilities CAPEX and COGS. This should facilitate decision making when assessing the risk and reward of each scenario.

The large-scale recombinant production of thaumatin can address the growing market need for natural, safe, non-caloric sweeteners. Like stevia, the advent of thaumatin as a sugar substitute is contingent on the feasibility of its large-scale manufacturing which was addressed in this work. However, there are also social, cultural, and behavioral factors impacting sugar consumption habits that were not considered. Consumer’s preference of such products will open the door for more plant-made biologics for food and beverage applications, which could drive the adoption of cost-effective solutions to rising challenges through environmentally friendly and sustainable processes.

## Figures and Tables

**Table 1:** Capital expenditure (CAPEX), annual operating costs (AOC), and cost of goods sold (COGS) for transgenic thaumatin production facilities. Depreciation is based on the 10-year straight line method.

Facility	Upstream (Field)	Upstream (Indoor)	Downstream (With Chromatography)
CAPEX ($ million)	10.2	186	115
AOC without depreciation ($ million)	3.59	96.1	25.0
COGS without depreciation ($/kg)	71.7	1920	499
AOC with depreciation ($ million)	4.22	110	35.3
COGS with depreciation ($/kg)	84.5	2200	706
	**Upstream** **(Field)**	**Upstream** **(Indoor)**	**Downstream** **(Without Chromatography)**
CAPEX ($ million)	9.22	157	83.7
AOC without depreciation ($ million)	3.00	80.3	12.8
COGS without depreciation ($/kg)	60.0	1600	258
AOC with depreciation ($ million)	3.56	94.3	20.4
COGS with depreciation ($/kg)	71.2	1890	408

**Table 2:** Direct fixed capital (DFC), capital expenditures (CAPEX), annual operating cost (AOC), and cost of goods sold (COGS) for spinach transient thaumatin production facility. Depreciation is based on the 10-year straight line method.

	Viral Particles Production	Spinach Growth	Downstream Processing	Working Capital, Start-Up, and Validation Costs	Total
DFC(section)/CAPEX(total)	2.53	9.74	64.4	4.49	81.2
($ million)	1.32	5.12	11.1	-	17.5
AOC without depreciation ($ million)	26.4	103	221	-	350
COGS without depreciation ($/kg)	1.56	5.38	17.2	-	24.1
AOC with depreciation	31.2	108	344	-	482

**Table 3:** Daily and annual amount of thaumatin required for each sugar-reduction scenario modeled in sweetened beverages. MT, metric ton.

Scenario	Thaumatin II Requirement (kg) Per 1 Billion 12 fl Oz Drinks (Per Day)	Annual Thaumatin Requirement (MT)
20% sugar reduced	460	170
30% sugar reduced	770	280
50% sugar reduced	1600	600

## Data Availability

This study is a techno-economic analysis of manufacturing processes for a biologic product and was conducted in part using the in-silico modeling software SuperPro Designer. The Base Case SuperPro model developed in this study is publicly available and can be downloaded from the following website: https://mcdonald-nandi.ech.ucdavis.edu/tools/techno-economics/. A free trial version of SuperPro (http://www.intelligen.com/demo.html) can be used to view the model.

## Supplementary Materials

The following are available online at https://www.mdpi.com/article/10.3390/foods10040838/s1, Figure S1: SuperPro Designer model flowsheet for vertical farming (indoor) upstream transgenic production facility, Figure S2: SuperPro Designer model flowsheet for thaumatin transient production in spinach, Table S1: Transgenic thaumatin production facilities base case design parameters and assumptions, Table S2: Downstream processing losses breakdown per unit operation, Table S3: Transient production of thaumatin in spinach base case parameters and assumptions, Table S4: Transgenic production facilities DFC estimation parameters, Table S5: Transient production facility DFC estimation parameters, Table S6: Working capital (WC) estimation parameters for all facilities, Table S7: Transgenic production facilities detailed annual labor cost, Table S8: Transient production facility detailed annual labor cost, Table S9: Transgenic production facilities dependent costs estimation parameters, Table S10: Transient production facilities dependent costs estimation parameters.
