# Supplementary material for: Process Simulation and Techno-Economic Analysis of Large-Scale Bioproduction of Sweet Protein Thaumatin II
Source: Foods. 2021 Apr 12;10(4):838. doi: 10.3390/foods10040838 (PMC8069865; doi:10.3390/foods10040838)
Supplement: Supplementary file 1 [file foods-10-00838-s001.pdf]

Supplementary material

Process Simulation and Techno-Economic Analysis of Large-Scale Bioproduction of Sweet Protein Thaumatin II

Kirolos D. Kelada[1], Daniel Tusé[2], Yuri Gleba[3], Karen A. McDonald[1,4] and Somen Nandi[1,4*]

[1]Department of Chemical Engineering, University of California, Davis, CA, USA; [2]DT/Consulting Group, Sacramento, CA, USA; [3]Nomad Bioscience GmbH, Halle, Germany; [4]Global HealthShare® Initiative, University of California, Davis, CA, USA

* Correspondence: author. Email: snandi@ucdavis.edu; Address: 1035 Academic Surge, 1 Shields Avenue, Davis, CA 95616

Transgenic Facilities Economic Inputs

The facilities' economic evaluation is based on the US dollar value in 2020. A 4% inflation rate is used to adjust for equipment purchase prices from previous years. Field growth economic variables were obtained from various sources. Fertilizer quantity and cost were obtained from a cost estimation spreadsheet developed University of Kentucky department of Agricultural Economics (UKAE) [1]. The quantity was estimated by linear extrapolation based on 120 days growth and adjusted from the 42 days growth period in this model. Field irrigation was estimated from the Food and Agriculture Organization of the United Nations water requirement for tobacco [2]. For land purchase prices, farm real estate average value per acre in Florida was estimated based on the USDA land values 2018 summary report [3]. Drip irrigation costs were obtained from [4]. Fuel, lubrication, and repair costs for tractors and other field equipment were obtained from the 2015 UC ANR field cost study [5] and were adjusted based on the average annual spinach producer price indices obtained from the Federal Reserve Bank of St. Louis [6]. Indoor cost variables were adapted from [7]. Downstream processing economic values were obtained from [8], [7], SuperPro Designer default values, and WPK. Startup and validation costs were estimated as 5% of direct fixed capital (DFC). Working capital was estimated to cover expenses for 30 days of operation.

Transient Facility Economic Inputs

The facility's economic evaluation is based on the US dollar value in 2020. A 4% inflation rate is used to adjust for equipment purchase prices from previous years. Spinach field growth economic parameters were adjusted based on the average annual spinach producer price indices obtained from the Federal Reserve Bank of St. Louis (FRED Federal Reserve Bank of St. Louis, 2020). VPL's economic parameters were adapted from [8] and equipment purchase prices were adjusted according to the following equation:

$$C = C_0 \left(\frac{Q}{Q_0}\right)^{0.6}$$

where C is the equipment cost, C_0 is the base cost, Q is the capacity variable, Q_0 is the base capacity. Other unit operation equipment costs were estimated from the built-in SuperPro Designer cost models.

Farm real estate average value per acre in California was estimated based on the USDA land values 2018 summary report (USDA, 2018). Drip irrigation costs were obtained from Simonne et al. (2008). Fuel, lubrication, and repair costs for tractors and other field equipment were obtained from the 2015 UC ANR cost study [5]. Downstream processing economic values were obtained from Nandi et al. (2016), McNulty et al. (2019), SuperPro Designer default values, and WPK.

Table 1. Transgenic thaumatin production facilities base case design parameters and assumptions; FW, fresh weight; WPK, working process knowledge; Calc, calculation; MT, metric ton.

Parameter	*Value*	*Unit*	*Reference*
Upstream facility (field), with downstream chromatography			
Production level	50	MT thaumatin/year	Assumption
Number of batches	157	batches/year	Calc
Batch duration	45.3	days	Calc
Recipe cycle time	2	days	Calc
Growth time (seeding to induction)	35	days	WPK
Incubation time (induction to harvest)	7	days	WPK
Land turnaround duration	3	days	WPK
Thaumatin expression level	1.5	g/kg FW	WPK
Plant density	130,000	plants/acre	Assumption (based on 3 plants/ft^2)
N. tabacum aerial biomass at harvest	100	g/plant	[9]
Germination efficiency	90	%	Assumption
Acreage per batch	24.5	acres	Calc
Number of plots	22	plots/total field	Calc
Total field acreage (footprint)	538	acres	Calc
Total annual cultivated acreage	3,850	acres/yr	Calc
Location	Florida, USA		Assumption
Upstream facility (indoor), with downstream chromatography			
Production level	50	MT thaumatin/yr	
Number of batches	157	batches/yr	Calc
Batch duration	42.6	days	Calc
Recipe cycle time	2	days	Calc
Growth time (seeding-induction)	35	days	[7]
Incubation time (induction-harvest)	7	days	[7]
Thaumatin expression level	1.5	g/kg FW	WPK
N. benthamiana aerial biomass at harvest	15	g/plant	[7]
Plants per batch	21,200,000	plants/batch	Calc
Germination efficiency	95	%	Assumption
Plants per tray	94	Plants tray	[7]
Tray area	0.15	m^2/tray	[7]
Growth space design	10	layers	Assumption
Growth space utilization	90	%	Assumption
Facility footprint	83,000	m^2	Calc
Downstream processing facility, with chromatography			
Batch duration	54.5	hours	Calc
Downstream recovery	66.8	%	Assumption
Final product purity	98.0	%	Assumption
Downstream processing facility, without chromatography			
Batch duration	38.4	hours	Calc
Downstream recovery	80	%	Assumption
Final product purity	74.8	%	Assumption

Table 2. Downstream processing losses breakdown per unit operation; P&F, plate and frame filtration; DSP, downstream processing; UF/DF, ultrafiltration/diafiltration; Chrom, chromatography.

DSP facility without chromatography							
Step	**Screw Press**	**P&F 1**	**P&F 2**	**P&F 3**	**UF/DF**	**Chrom and UF/DF 2**	Drying
Loss (% of initial thaumatin)	3.0	5.0	5.0	1.5	5.0	-	0.5
Cumulative recovery (% of initial thaumatin)	97.0	92.0	87.0	85.5	80.5	-	80.0
Start (kg/batch)	398	386	366	346	340	-	320
End (kg/batch)	386	366	346	340	320	-	319
% loss per unit	3.0	5.2	5.4	1.7	5.8	-	0.6
DSP facility with chromatography							
Step	Screw Press	P&F 1	P&F 2	P&F 3	UF/DF	Chrom and UF/DF 2	Drying
Loss (% of initial thaumatin)	3.0	5.0	5.0	1.5	5.0	13.2	0.5
Cumulative recovery (% of initial thaumatin)	97.0	92.0	87.0	85.5	80.5	67.3	66.8
Start (kg/batch)	477	463	439	415	408	384	320
End (kg/batch)	463	439	415	408	384	320	319
% loss per unit	3.0	5.2	5.4	1.7	5.8	17	0.6

Table 3. Transient production of thaumatin in spinach base case parameters and assumptions FW, fresh weight; WPK, working process knowledge; Calc, calculation; MT, metric ton.

Parameter	*Value*	*Unit*	*Reference*
Overall facility			
Production level	50	MT thaumatin/yr	
Number of batches	153	batches/yr	Calc
Batch duration	68	days	Calc
Recipe cycle time	1.94	days	Calc
Location	California, USA		
Spinach field growth			
Growth time (seeding-spraying)	45	days	[10]
Incubation time (spraying-harvest)	15	days	WPK
Thaumatin expression level	1	g/kg FW	WPK
Field plant density	174,240	plants/acre	Assumption
Spinach yield	15,240	kg FW/acre	[10], WPK
Seed quantity	1.25 million	seeds/acre	[10]
	31.3	lbs/acre	[5]
Acreage per batch	22.6	acres/batch	Calc
Number of plots	34	plots/total field	Calc
Total field acreage (footprint)	767	acres	Calc
Total cultivated acreage (assuming no reusing of land)	3,450	acres	Calc
Viral particles production			
N. benthamiana growth time	35	days	[8]

(seeding-infiltration)			
N. benthamiana incubation time (infiltration-harvest)	7	days	[8]
Viral particles expression level	1	g/kg FW	[11]
Viral particle concentration in spray suspension	10^{14}	particles/L	WPK
Viral particle molecular weight	31,750	kDa	WPK
Spray volume requirement	2	mL/plant	WPK
	Downstream Processing		
Downstream recovery	95	%	WPK
Downstream Processing time	30.2	hrs/batch	Calc
Final thaumatin purity	94	%	Assumption

Table 4. Transgenic production facilities DFC estimation parameters. DFC, direct fixed cost; PC, purchase cost; DC, direct cost; IC, indirect cost; OC, other costs.

<table>
<tr><th></th><th>Upstream (Field)</th><th>Upstream (Indoor)</th><th>Downstream</th></tr>
<tr><td rowspan="5">Unlisted Equipment</td><td>Seeding:
0.03 x PC*</td><td>Seeding:
0.2 x PC</td><td rowspan="5">Entire Facility:
0.2 x PC</td></tr>
<tr><td>Plant Growth:
0.03 x PC</td><td>Plant Growth:
0.2 x PC</td></tr>
<tr><td>Induction + Incubation:
0.03 x PC</td><td>Induction + Incubation:
0.2 x PC</td></tr>
<tr><td>Harvesting:
0.03 x PC</td><td>Harvesting
0.2 x PC</td></tr>
<tr><td>Transportation:
0.2 x PC</td><td>Transportation
0.2 x PC</td></tr>
<tr><td rowspan="5">Lang Factor</td><td>Seeding:
1.0 x PC</td><td>Seeding:
3.0 x PC</td><td>Entire Facility:
DFC= DC+IC+OC</td></tr>
<tr><td>Plant Growth:
1.0 x PC</td><td>Plant Growth:
3.0 x PC</td><td>DC: **
Piping (A)= 0.35 x PC
Instrumentation (B)= 0.40 x PC
Insulation (C)= 0.03 x PC
Electrical Facilities (D)= 0.10 x PC
Buildings (E)= 0.45 x PC
Yard Improvement (F)= 0.15 x PC
Auxiliary Facilities (G)= 0.40 x PC</td></tr>
<tr><td>Induction + Incubation:
1.0 x PC</td><td>Induction + Incubation:
3.0 x PC</td><td>Unlisted Equipment Installation Cost= 0.50 x Unlisted Equipment purchase cost
Listed Equipment Installation Cost: Equipment specific</td></tr>
<tr><td>Harvesting:
1.0 x PC</td><td>Harvesting:
3.0 x PC</td><td>IC:
Engineering= 0.25 x DC
Construction= 0.25 x DC</td></tr>
<tr><td>Transportation:
3.0 x PC</td><td>Transportation:
3.0 x PC</td><td>OC:
Contractor's Fee= 0.05 x (DC + IC)
Contingency= 0.10 x (DC + IC)</td></tr>
</table>

*Purchase Cost (PC) = Listed equipment purchase cost + unlisted equipment purchase cost

** Direct Cost (DC)= PC + Installation +A + B + C + D + E + F + G.

Table 5. Transient production facility DFC estimation parameters. DFC, direct fixed cost; PC, purchase cost; DC, direct cost; IC, indirect cost; OC, other costs; VPL, virion production laboratory.

	VPL	*Field Growth*	*Downstream*
Unlisted Equipment	0.2 x PC	0.03 x PC	Entire Facility:<br>0.2 x PC
Lang Factor	3.0 x PC	1.0 x PC	Entire Facility:<br>DFC= DC+IC+OC<br>DC: **<br>Piping (A)= 0.35 x PC<br>Instrumentation (B)= 0.40 x PC<br>Insulation (C)= 0.03 x PC<br>Electrical Facilities (D)= 0.10 x PC<br>Buildings (E)= 0.45 x PC<br>Yard Improvement (F)= 0.15 x PC<br>Auxiliary Facilities (G)= 0.40 x PC<br>Unlisted Equipment Installation Cost= 0.50 x Unlisted Equipment purchase cost<br>Listed Equipment Installation Cost: Equipment specific<br>IC:<br>Engineering= 0.25 x DC<br>Construction= 0.25 x DC<br>OC:<br>Contractor’s Fee= 0.05 x (DC + IC)<br>Contingency= 0.10 x (DC + IC)

*Purchase Cost (PC) = Listed equipment purchase cost + unlisted equipment purchase cost.

** Direct Cost (DC)= PC + Installation +A + B + C + D + E + F + G.

Table 6. Working capital (WC) estimation parameters for all facilities. .

Parameter	*Value*
Cover labor expenses for	30 days
Cover raw materials expenses for	30 days
Cover utilities expenses for	30 days
Cover waste treatment expenses for	30 days
Startup and Validation	5% of DFC

Table 7. Transgenic production facilities detailed annual labor cost. BLC, basic labor cost; TLC, total labor cost.

Facility	*Labor type*	*BLC*	*TLC****	*Direct Demand Hours per year*	*Total Demand Hours per year*
Upstream (field)	Upstream operator	$17/h	$39.10/h	30,647	40,863
Upstream (Indoor)	Upstream operator	$20/h	$46/h	3,938	4,145
Downstream	Downstream operator	$25/h	$57.50/h	21,663	28,884

***TLC= BLC x (1 + Benefits (0.4) + Supervision (0.2) + Supplies (0.1) + Administration (0.6)).

Table 8. Transient production facility detailed annual labor cost. BLC, basic labor cost; TLC, total labor cost.

Facility Section	*Labor type*	*BLC*	*TLC****	*Direct Demand Hours per year*	*Total Demand Hours per year*
VLP	Upstream operator	$20/h	$46/h	13,616	18,155
Field Growth	Field operator	$17/h	$39.10/h	36,620	48,827
Downstream	Downstream operator	$25/h	$57.50/h	7,919	10,559

***TLC= BLC x (1 + Benefits (0.4) + Supervision (0.2) + Supplies (0.1) + Administration (0.6)).

Table 9. Transgenic production facilities dependent costs estimation parameters.

Facility	Raw Material	Unit Cost
Upstream (field)	Maintenance	Included as consumables
	Depreciation	Straight line over 10 years (5 % salvage value). Land is non-depreciable.
	Insurance	0.09% DFC
	Local taxes	2.51% DFC
	Factory expenses	0.12% DFC
Upstream (indoor)	Maintenance	Section dependent (0.10-0.40 % DFC)
	Depreciation	Straight line over 10 years (5 % salvage value)
	Insurance	1% DFC
	Local taxes	2% DFC
	Factory expenses	5% DFC
Downstream	Maintenance	Equipment specific
	Depreciation	Straight line over 20 years (5 % salvage value)
	Insurance	1% DFC
	Local taxes	2% DFC
	Factory expenses	5% DFC

Table 10. Transient production facilities dependent costs estimation parameters.

Facility	Raw Material	Unit Cost
VPL	Maintenance	0.40 % DFC
	Depreciation	Straight line over 10 years (5 % salvage value). Land is non-depreciable.
	Insurance	0.09% DFC
	Local taxes	2.51% DFC
	Factory expenses	0.12% DFC
Field growth	Maintenance	Included as consumables
	Depreciation	Straight line over 10 years (5 % salvage value)
	Insurance	0.09% DFC
	Local taxes	2.51% DFC
	Factory expenses	0.12% DFC
Downstream	Maintenance	Equipment specific
	Depreciation	Straight line over 10 years (5 % salvage value)
	Insurance	1% DFC
	Local taxes	2% DFC
	Factory expenses	5% DFC

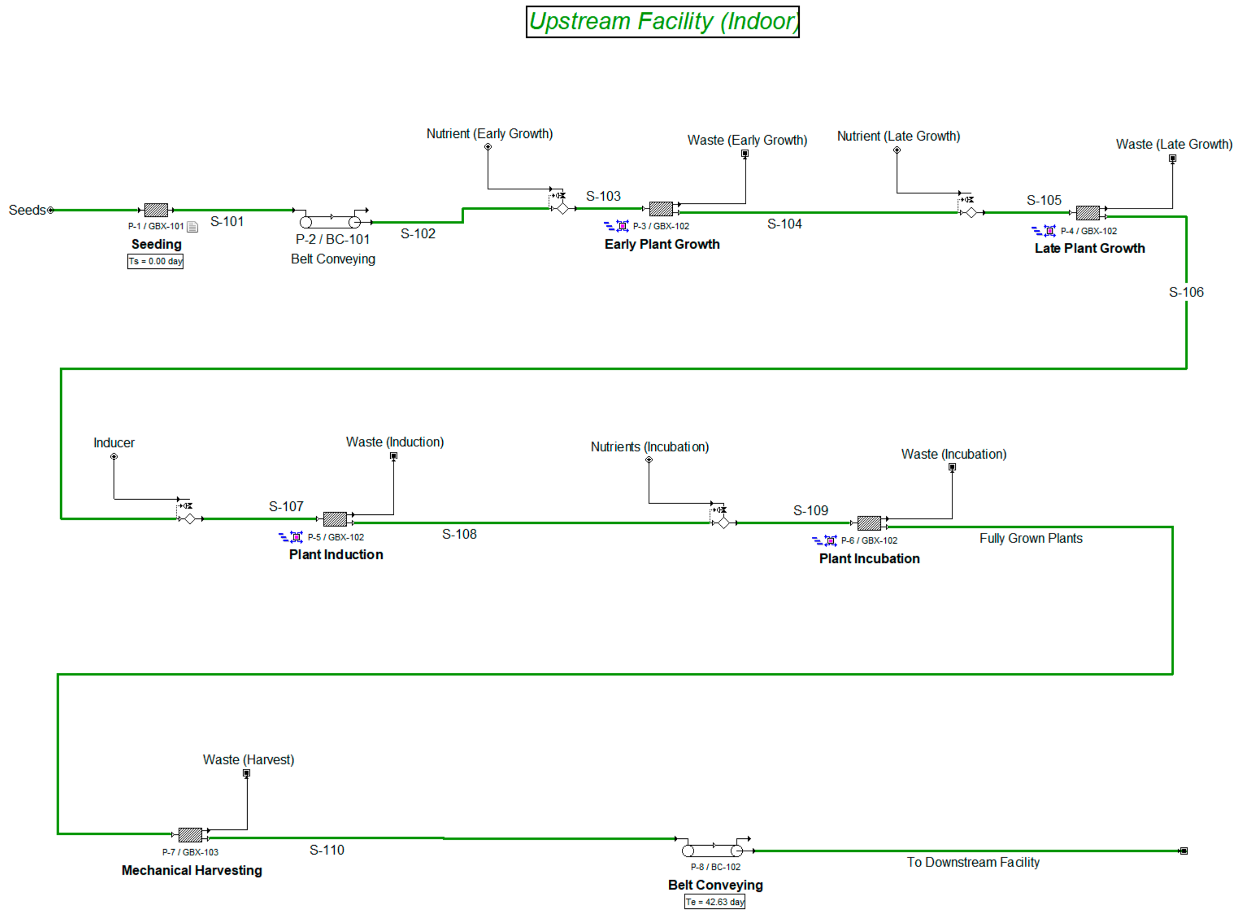

Figure S1. SuperPro Designer model flowsheet for vertical farming (indoor) upstream transgenic production facility

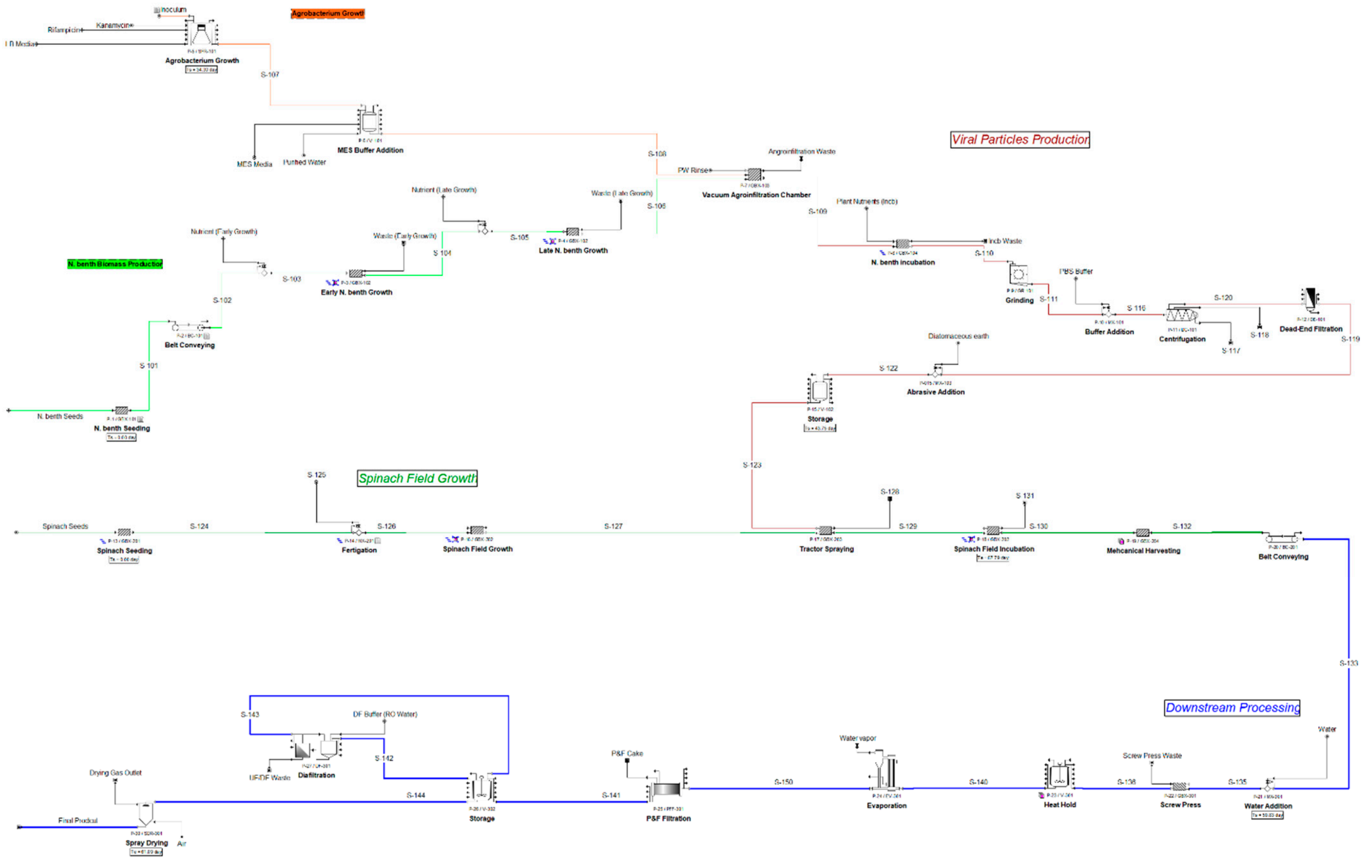

Figure S2. SuperPro Designer model flowsheet for thaumatin transient production in spinach. V-103: 73,000L (10 in parallel)

Supplemental Materials References:

1. TN-KY Burley Tobacco Budget Burley Tobacco Budget 2018 Estimated Costs And Returns Available online: http://www.uky.edu/Ag/AgriculturalEconomics/pubs/exttobbudget201857.xls (accessed on Mar 15, 2019).
2. Food and Agriculture Organization of the United Nations Land & Water Tobacco Available online: http://www.fao.org/land-water/databases-and-software/crop-information/tobacco/en/ (accessed on Mar 15, 2019).
3. United States Department of National Agriculture (USDA) Agricultural Statistics Service *Land Values 2018 Summary*; **2018**.
4. Simonne, E.; Hochmuth, R.; Breman, J.; Lamont, W.; Treadwell, D.; Gazula, A. Drip-irrigation systems for small conventional vegetable farms and organic vegetable farms. *University of Florida IFAS Extension* **2008**.
5. Smith, R.F.; Tumber, K.P. Sample Costs to Produce and Harvest Organic Spinach; **2015**.
6. FRED Federal Reserve Bank of St. Louis U.S. Bureau of Labor Statistics, Producer Price Index by Commodity for Farm Products: Spinach [WPU01130224] Available online: https://fred.stlouisfed.org/series/WPU01130224 (accessed on Apr 23, 2020).
7. McNulty, M.J.; Gleba, Y.; Tusé, D.; Hahn-Löbmann, S.; Giritch, A.; Nandi, S.; McDonald, K.A. Techno-economic analysis of a plant-based platform for manufacturing antimicrobial proteins for food safety. *Biotechnology Progress* **2019**, *36*, e2896, doi:10.1002/btpr.2896.
8. Nandi, S.; Kwong, A.T.; Holtz, B.R.; Erwin, R.L.; Marcel, S.; McDonald, K.A. Techno-economic analysis of a transient plant-based platform for monoclonal antibody production. *mAbs* **2016**, *8*, 1456–1466, doi:10.1080/19420862.2016.1227901.
9. Knödler, M.; Rühl, C.; Emonts, J.; Buyel, J.F. Seasonal Weather Changes Affect the Yield and Quality of Recombinant Proteins Produced in Transgenic Tobacco Plants in a Greenhouse Setting. *Frontiers in Plant Science* **2019**, *10*, 1245, doi:10.3389/fpls.2019.01245.
10. Koike, S.T.; Cahn, M.; Cantwell, M.; Fennimore, S.; Lestrange, M.; Natwick, E.; Smith, R.F.; Takele, E. Spinach production in California. **2011**.
11. Klimyuk, V.; Pogue, G.; Herz, S.; Butler, J.; Haydon, H. Production of Recombinant Antigens and Antibodies in Nicotiana benthamiana Using `Magnifection' Technology: GMP-Compliant Facilities for Small- and Large-Scale Manufacturing. In *Plant Viral Vectors*; Palmer, K., Gleba, Y., Eds.; Springer Berlin Heidelberg: Berlin, Heidelberg, 2014; pp. 127–154 ISBN 978-3-642-40829-8.
